# A Systematic Approach for Developing 3D High-Quality PDMS Microfluidic Chips Based on Micromilling Technology

**DOI:** 10.3390/mi13010006

**Published:** 2021-12-22

**Authors:** Amin Javidanbardan, Ana M. Azevedo, Virginia Chu, João P. Conde

**Affiliations:** 1 IBB—Institute for Bioengineering and Biosciences, Instituto Superior Técnico, Universidade de Lisboa, 1049-001 Lisboa, Portugal; amin.javidanbardan@tecnico.ulisboa.pt; 2 Department of Bioengineering, Instituto Superior Técnico, Universidade de Lisboa, 1049-001 Lisboa, Portugal; 3 Instituto de Engenharia de Sistemas e Computadores—Microsistemas e Nanotecnologias (INESC MN), 1000-029 Lisboa, Portugal; vchu@inesc-mn.pt

**Keywords:** micromachining strategies, micro/mesoscale milling, 3D microfluidic structure, PMMA, PDMS, surface quality, optimization, double casting, sensor integration, microsystem integration

## Abstract

In recent years, there has been an increased interest in exploring the potential of micro-and mesoscale milling technologies for developing cost-effective microfluidic systems with high design flexibility and a rapid microfabrication process that does not require a cleanroom. Nevertheless, the number of current studies aiming to fully understand and establish the benefits of this technique in developing high-quality microsystems with simple integrability is still limited. In the first part of this study, we define a systematic and adaptable strategy for developing high-quality poly(methyl methacrylate) (PMMA)-based micromilled structures. A case study of the average surface roughness (*Ra*) minimization of a cuboid column is presented to better illustrate some of the developed strategies. In this example, the *Ra* of a cuboid column was reduced from 1.68 μm to 0.223 μm by implementing milling optimization and postprocessing steps. In the second part of this paper, new strategies for developing a 3D microsystem were introduced by using a specifically designed negative PMMA master mold for polydimethylsiloxane (PDMS) double-casting prototyping. The reported results in this study demonstrate the robustness of the proposed approach for developing microfluidic structures with high surface quality and structural integrability in a reasonable amount of time.

## 1. Introduction

Over the past three decades, there has been sustained research activity in developing microfluidic systems [1]. This field is a growing and competitive area of research due to the wide application of miniaturized systems in different fields, including biomedical, pharmaceutical, environmental, and chemical engineering [2,3]. So far, various microfabrication methods have been suggested to develop microfluidic structures, each having its own relative pros and cons [4,5].

In recent years, there has been great interest in exploring cost-effective and rapid microfabrication technologies to reduce the high capital and operation expenses, namely when cleanroom microfabrication is involved [4,6]. Computer numerical control (CNC) micro/mesoscale milling is a promising microfabrication method based on subtractive processes on workpiece materials using miniaturized cutting tools [7,8,9]. Among various workpiece materials, thermoplastics, such as polystyrene, polycarbonate, and PMMA, are popular choices for micromilling due to their low cost, low density, and good machinability. Several studies have explored the suitability of micromilled plastics in developing lab-on-a-chip systems for miniaturized cell cultivation and bioanalysis [4]. Compared to conventional microfabrication methods such as soft lithography, this technique offers various advantages, including fast and semi-continuous operation, high design flexibility, and low capital and maintenance costs [2,6].

Several studies reported the successful use of micromilling technology to directly fabricate microfluidic structures from thermoplastics [10]. Nevertheless, PDMS elastomer offers better material compatibility and easier process integration and operation with fewer problems related to fluid leakage. As a result, some studies have implemented micromilled structures as a master mold with positive features for PDMS patterning [11,12]. This type of master mold suffers from limitations, such as limited channel and chamber shape options and the requirement for the fine milling of large surface areas. Alternatively, studies have reported using intermediate molds to create a final PDMS structure from the original master mold with negative features [13,14]. However, there are relatively few of these studies, and various possibilities for making high-quality 3D PDMS microstructures based on micromachining are still unexplored. In addition, except for a few studies [4,15], most research work concerning the micromachining process has focused primarily on examining the mechanism and conditions of the milling process or defining specific strategies for developing microstructures with a particular application.

This study combines both aspects in developing micromilling-based structures. In the first section of this study, we define a systematic strategy for developing a high-quality PMMA-based microfluidic system based on in-house experimental findings and published literature. This section presents a case study of average surface roughness (*Ra*) minimization of a cuboid column to illustrate the strategy used in process optimization, based on response surface methodology (RSM) and a postprocessing step. In the second part of this study, we introduced a specific design strategy for developing a 3D PDMS microfluidic system from micromilled PMMA with negative features, which offers the possibility of circular channel creation and sensor integration.

## 2. Micromachining Setup

**Figure 1:**
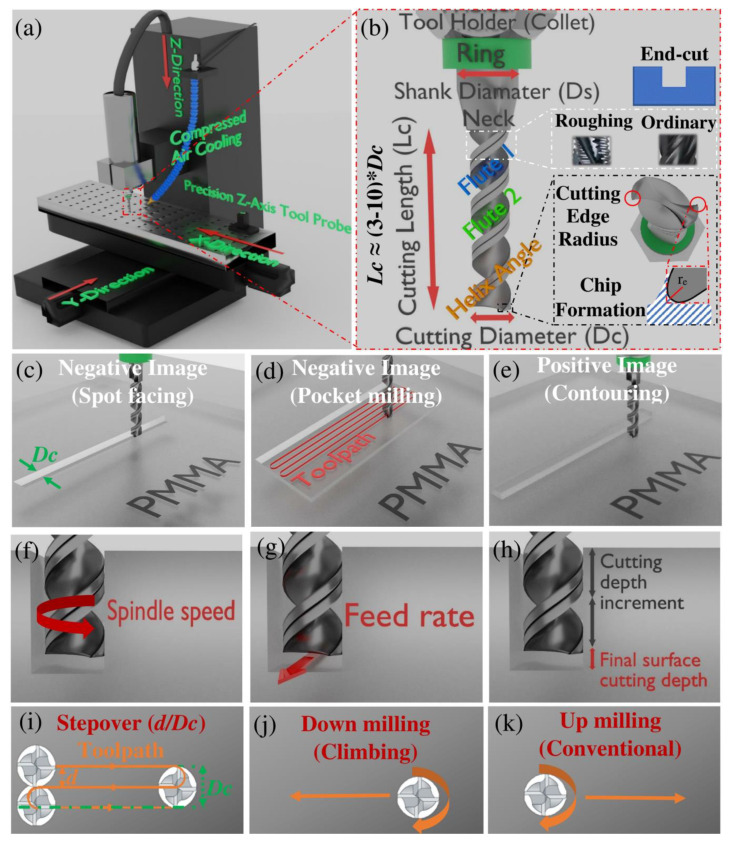
Schematic illustration of (**a**) 3-axis CNC micromilling machine setup; (**b**) Endmill cutting tool characteristics; (**c**) Spot facing; (**d**) Pocket milling; (**e**) Contouring (**f**); Spindle speed parameter; (**g**) Feed rate parameter; (**h**) Cutting depth parameter; (**i**) Stepover concept; (**j**) Climb milling; (**k**) Conventional milling. Straight arrows represent the direction of travel, and spin arrows show the direction of rotation.

The versatility of current micromachining systems has made it challenging to create a unified methodology for fabricating reproducible microfluidic structures. Currently, depending on the system accuracy (anywhere from 1 μm up to 100 μm in the *z*-direction, the direction is shown in Figure 1a) and the degree of automation in the micromilling system (such as the presence of an automatic tool changer), the price of these machines varies from $15,000 to $220,000 [4]. This study focuses on using a simple and cost-effective micromilling system to develop high-quality PMMA microstructures. This section presents brief background information regarding the main components of standard micromilling systems and cutting tools. In addition, the design and development steps for creating microstructures with commonly used micromachining operations are briefly described.

### 2.1. Main Equipment

Figure 1a shows the schematic view of the implemented 3-axis micromilling equipment under dry machining. The characteristics of the cutting tool, in this case, endmill, are illustrated in Figure 1b and will be discussed in detail in Section 3. In this technique, the proper material removal is performed by chip formation (Figure 1b) and removal from the workpiece without deformation.

### 2.2. Design Procedure

The geometrical design of the microstructure is established using a computer-aided design (CAD) system, which in this study was AutoCAD 2020 (Autodesk, San Rafael, CA, USA). CamBam software was implemented in this study to define the type of micromachining operation and toolpath (see Section 2.3) on the CAD source file and create the respective computer-aided manufacturing (CAM) files (GCode). After creating the milling operation details, the software estimates the total machining time (*t*). The CAM file is then transferred to Machine Controller software (Mach 3) to initiate the micromilling process.

### 2.3. Machining Operations

Various types of endmill processing have been described for both macro- and micromachining operations. The most important and relevant processes for developing microfluidic structures with endmills are presented in Figure 1c–e. As shown in Figure 1c, spot facing is a negative feature design in which an internal channel or hole is fabricated on the surface of the workpiece with a width equal to the diameter of the endmill (*D_c_*). Pocket milling is another type of negative feature structure (Figure 1d) in which the surface’s width and length are wider than endmill diameter. Therefore, multidirectional toolpaths should be performed. Figure 1e illustrates positive feature processing, the inverse of pocket milling, called contouring. Face milling is another type of endmill processing in which the top layer of the workpiece is machined entirely in a constant depth value, without creating any features in the process.

## 3. Design Methodology for Developing Micromilling-Based Systems

**Figure 2:**
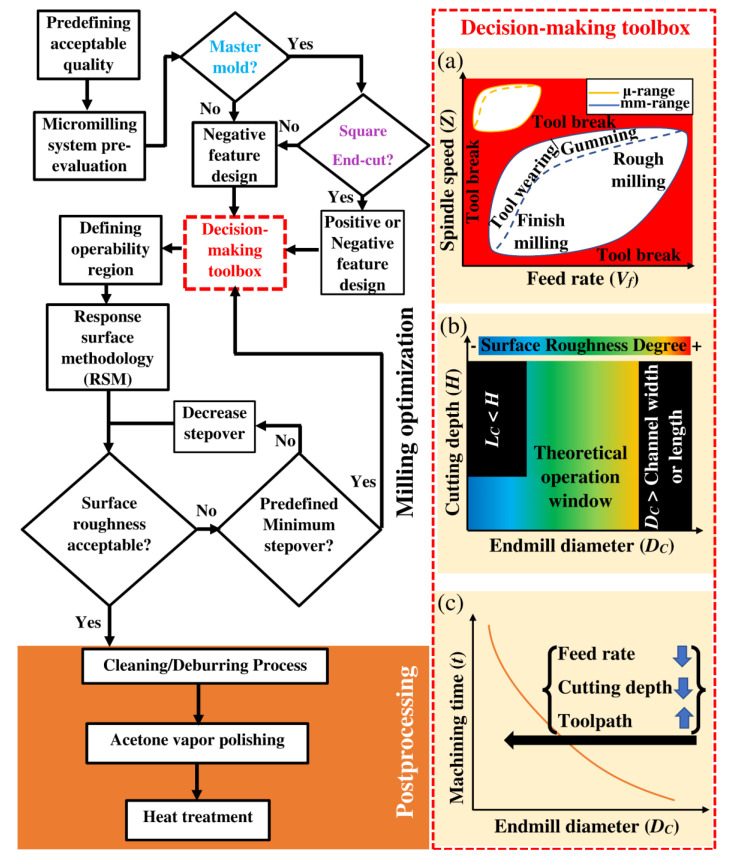
A proposed systematic strategy for developing high-quality micromilling-based microfluidics. (**a**) Schematic diagram showing operation window based on feed rate and spindle speed for endmills with microscale and milliscale cutting diameter; (**b**) Schematic diagram demonstrating the operation window and surface roughness degree based on cutting diameter of endmill; (**c**) Schematic diagram showing the relationship between machining time and endmill cutting diameter.

The wide variation in size, shape, and materials of micromilling tools and workpieces requires collecting a significant amount of data to define the best operating condition for each scenario [4,16]. Despite the similarity between macromilling and micromilling in terms of physical appearance, the underlying cutting mechanism is different between these two systems [17,18]. Hence, the guidelines for macro-scale systems cannot be transferred directly to microscale systems, and new protocols should be developed for fabricating microfluidic systems with acceptable pertinent qualities [8,18,19]. In this section, we developed a holistic and simple strategy for creating micromilling-based microfluidic systems based on previously reported data, in-house protocols, and optimization experiments. Figure 2 illustrates the methodology for developing and improving the quality of micro-and mesoscale structures using micromilling technology for thermoplastics- PMMA in this study. Detailed information relating to the flowchart (Figure 2) is provided in this section and partly in Section 4.

### 3.1. Pre-Defining Acceptable Quality Criteria

The quality of the micromilled structure can be mainly defined based on the presence of damage, surface roughness, size accuracy, and precision [2,4,7,20,21].

#### 3.1.1. Size Accuracy and Precision

The required size accuracy of a microstructure is one of the main parameters which defines whether the respective micromilling technology is a suitable microfabrication technique for the target microstructure. Generally, the resolution of the milled microstructure should mainly depend on the micromilling system’s accuracy [4]. Nevertheless, other factors could negatively impact the size accuracy and precision, especially in the *z*-direction (see Section 3.2). For less sophisticated micromilling systems, dimensions smaller than 100 μm are not recommended [4].

#### 3.1.2. Damage Mechanisms

Various damage mechanisms, including burr formation, edge overcut, internal surface damage, and channel clogging, could be present in the micromilled structures [2,7]. Each damage mechanism has been extensively described by Walsh et al. [7]. Burr formation has been the most studied aspect and is in general less frequent in PMMA structures than in metals and ductile plastics, such as polypropylene, polycarbonate, polytetrafluoroethylene, and cyclic olefin copolymer [4,19,22,23].

#### 3.1.3. Surface Roughness

It is essential to pre-define the minimum acceptable surface roughness to meet the required characteristics for the specified microstructure. Transparency for imaging-based applications [6,24], cell–surface interaction [6], and bonding and sealing performance [24] are some of the characteristics which are mainly affected by surface roughness.

### 3.2. Pre-Evaluation of Micromachining Process

It is critical to initially check the following conditions in the micromilling system to improve process reliability and minimize the possibility of inconsistent results during the optimization and microfabrication process.

#### 3.2.1. Correct Alignment of the Worktable

The correct alignment of the micromachining worktable should be checked after setting up the micromachine and after any repair or upgrading of the setup [25]. Misalignment of the worktable would negatively impact the size accuracy in the *z*-direction.

#### 3.2.2. Endmill Sharpness and Cleanliness

Tool wearing, cutting edge (Figure 1b) rounding, or breaking have been reported as the main factors that can negatively affect accuracy and surface roughness [2,8,17]. The lifetime of the endmill is determined by the development of tool wearing, which mainly depends on load removal and cutting force [8,26,27]. Thus, before micromilling, it is advisable to visually inspect the presence of tool wear or use new endmills. The cleanliness of the endmill tool is another parameter that should be checked since the attachment of any material to the endmill can increase the surface roughness [25]. After performing the milling process, ultrasonication of endmill tools in acetone is recommended for polishing [25].

#### 3.2.3. Positioning and Consistency of Air Cooling

In dry machining, proper positioning of the air-cooling tube with constant air pressure should be ensured for the efficient removal of chips and avoidance of local heat accumulation, which leads to PMMA melting and negatively affects all the defined quality criteria [28].

#### 3.2.4. Workpiece Fixture

Different strategies for workpiece fixtures have been discussed extensively by Guckenberger et al. [4]. Poor workpiece setup could cause displacement, bending, or high vibration of the workpiece during micromilling, which decreases the accuracy and precision of micromachining [4]. Using double-sided adhesive tape or a vacuum chuck to fix the workpiece on the worktable are some simple approaches to avoid such problems [4].

### 3.3. Master Mold or Direct Microstructure

As demonstrated in the flowchart in Figure 2, it is essential to clarify if the PMMA will be used as a master mold or not for two main reasons. Firstly, the PMMA structure is incompatible with ketones, esters, strong bases, and oxidizing acids. Thus, implementing a micromilled PMMA structure as a microfluidic material is not possible when using these chemicals. Secondly, the micromachining time becomes an important parameter for the direct PMMA microfluidic system since the whole fabrication process should be repeated to reproduce each desired microstructure. As a result, the time parameter should be included in the optimization process as one of the target parameters to avoid long operation times. In the case of fabricating a master mold, the machining time does not pose such a dominant influence since it is not a repeated operation. Nonetheless, a shorter operation time offers the accelerated development of a multitude of prototypes for testing and optimization.

To develop direct microfluidic structures with different channel shapes, non-cuboidal, other types of endmill tools, such as ball nose, bull nose, and tapered endmills, are mainly used. If the micromilled structure was developed as a master mold, a negative image PMMA master mold should be fabricated for this purpose (see the flowchart in Figure 2). This master mold can then be employed to mold a positive master mold by using materials such as Polyvinyl siloxane (PVS) [29], epoxy adhesive [14], poly(ethylene terephthalate) (PET) [13], or PDMS [15]. As a result, the final replicas from the second master mold include channels and chambers with different shapes.

### 3.4. Decision-Making Toolbox

#### 3.4.1. Endmill Material

For micromilling plastic workpieces, uncoated tungsten carbide is generally acceptable for the end mill [4]. The single-crystal diamond micro-endmill is another material reported for the rapid and accurate micromilling of PMMA [30].

#### 3.4.2. Flute Number and Helix Angle

Endmills with two or four flutes (Figure 1b) are generally used for micromilling [4]. In theory, as presented in Equation (1), 4-flute endmills create lower surface roughness by reducing feed per tooth value (*f_z_*), causing smaller amplitude features on the surface [1]. In Equation (1), *Z*, *V_f_*, and *N* are spindle speed (Figure 1f), feed rate (Figure 1g), and flute number, respectively.

(1) $$f_{z}=\frac{V_{f}}{NZ}$$

On the other hand, 2-flute endmills offer added chip removal and can operate at higher spindle speeds [4]. Thus, increasing flutes from 2 to 4 might not significantly improve surface roughness at the lower spindle speeds. For milling plastics with milling areas in close proximity to each other, using a 2-flute endmill is recommended for better chip removal [25].

Figure 1b shows that the helix angle is another parameter for the milling tools. The helix angle of 30° is commonly used for the efficient micro-cutting and evacuation of plastic chips to avoid heat build-up [4,8].

#### 3.4.3. Aspect Ratio

The usual aspect ratio between the cutting length (*L_c_*) to the cutting diameter (*D_c_*) for micro-endmills is 3 (Figure 1b). Still, aspect ratios as high as 5 and 10 are also available for endmills with *D_c_* ≤ 100 μm and larger cutting diameters, respectively [9,22]. It should be noted, however, that in the high aspect ratio micro-endmill, the deflection (*δ*) is notably high (Equation (2)), which could negatively influence the precision and surface roughness [8]. *L_extra_* in Equation (2) represents the length of the endmill outside of the collet minus cutting length. In this equation, *E* and *F* are Young’s modulus and force, respectively.

(2) $$\delta =\frac{64F{\left(L_{c}+L_{extra}\right)}^{3}}{3ED_{c}{}^{4}}$$

#### 3.4.4. Cutting Diameter (*D_c_*)

With the current technology, microendmills with a cutting diameter as small as 5 μm are available on the market [31]. It is generally accepted that the surface roughness would steadily improve with a decrease in the cutting diameter (Figure 2b) [22,32]. It should be noted, however, that under the following conditions selecting endmills with a smaller cutting diameter is not advisable:

1. As demonstrated in Figure 2b, if the channel depth or height of the contour feature is larger than the cutting length (*L_c_*) of the endmill. Under this condition, given no extra space between the neck and cutting region, the neck (Figure 1b) would hit the sidewalls and damage the channel structure.
2. When a non-squared endmill is used in 3-axis micromachining devices for creating circular or tapered channels. In micromilling systems with more than 3 axes, although 3D-profile milling is possible, reaching acceptable surface roughness requires a time-intensive operation.
3. When the surface removal area/volume for the milling process is notably large. In this situation, reducing endmill size increases the operation time significantly not only by increasing the toolpaths but also by the requirement for reducing the feed rate and depth increment to avoid tool break (Figure 2c).

Besides these constraints, as shown in Figure 2a, the operability window for endmills with smaller diameters (e.g., 0.1–0.3 mm) is significantly smaller than larger endmills (e.g., 1–3 mm). Thus, the possibility of tool breakage or rounding and breaking of the cutting edge is higher during the optimization process, increasing the required time and cost for process optimization.

#### 3.4.5. Feed Rate, Spindle Speed and Cutting Depth

The typical parameters used to optimize the micromilling process are feed rate, spindle speed, and cutting depth [1,33,34,35,36,37,38]. The surface roughness is theoretically expected to be reduced by decreasing the feed rate and increasing the spindle speed via reducing the feed per tooth (see Equation (1)). Nonetheless, different studies have reported inconsistent results at high spindle speeds [4,23]. For the cutting depth parameter, previous studies have yielded inconclusive results regarding its impact [4].

The schematic operability window based on spindle speed and feed rate is shown in Figure 2a. The primary consideration for defining the operating range for the micromilling process is the avoidance of endmill tool breakage. Tool breakage can result from excessive cutting force or high frictional heat build-up, causing plastic melting and tool encasement. As shown in Figure 2a, other phenomena, such as tool wearing and the build-up of workpiece material on the tool, i.e., gumming, could also occur without tool breakage. This could adversely impact the micromilling quality by changing the effective size of the tool, variation of surface roughness, and creation of different types of damage along the single channel.

Two regions for roughing and finishing have been demonstrated in Figure 2a. In the roughing step, the primary strategy is to remove the initial bulk material of the workpiece as quickly as possible without causing significant damage to the endmill tool. Endmills with roughing teeth (Figure 1b) are suitable tools for this step. The final finishing step should be performed with the highest care based on the approach described in this paper.

#### 3.4.6. Stepover and Milling Direction

The stepover can be defined by the ratio of toolpath space of the tool during operation to its cutting diameter (Figure 1i). It has been reported that decreasing the stepover ratio could significantly improve surface roughness [26,32]. Nonetheless, it should be noted that the machining operation is increased by reducing the stepover ratio (*d*/*D_c_*) according to Equation (3). Thus, it is advisable to define a minimum limit for the stepover ratio to avoid a significant increase in operation time.

(3) $$t=\overset{h=n}{\underset{h=1}{\sum }}\frac{D_{c}}{{\left(d\right)}_{h}}*t_{h}$$

In Equation (3), *t_h_* represents machining time in each cutting depth with a stepover value of 1. Total operation time and the total number of cutting depth steps are represented by *t* and *n*, respectively.

Regarding the milling direction parameter, it has been reported that for the PMMA workpiece, the climb (down) milling direction (Figure 1j) could provide better surface roughness compared to the conventional (up) milling (Figure 1k) [3,32].

## 4. Results

### 4.1. Case Study of Surface Quality Improvement

This section presents a case study of *Ra* minimization of a cuboid column by using micromachining optimization based on RSM and postprocessing.

#### 4.1.1. Micromilling Optimization

**Figure 3:**
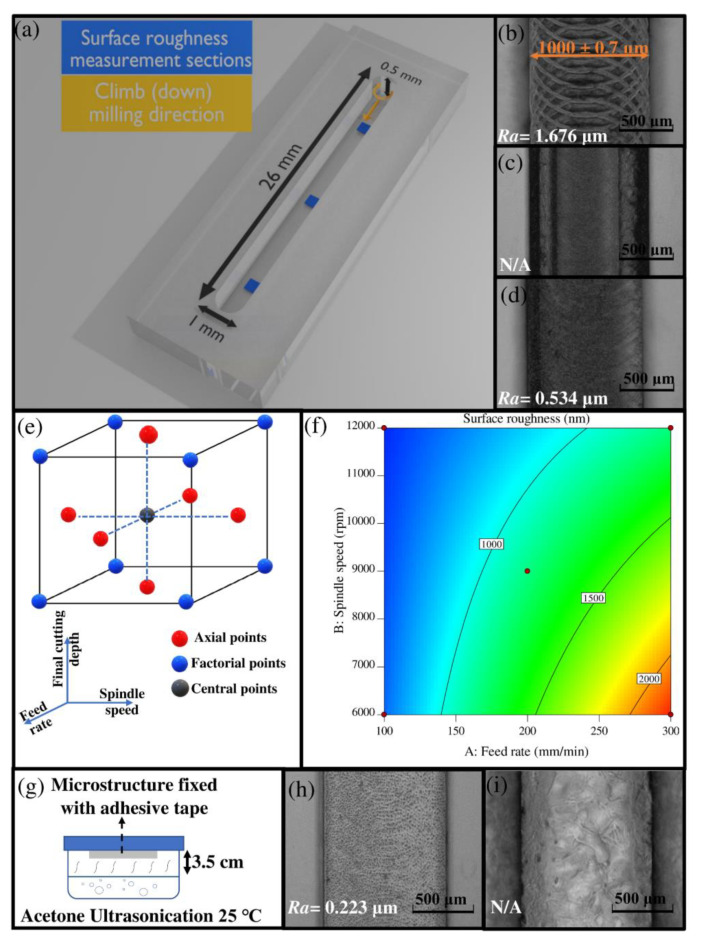
(**a**) Schematic representation of the model microfluidic column in the current case study; (**b**) Photograph of bottom surface finished by 1 mm endmill based on in-house protocol; (**c**) Photograph of the bottom surface finished by 1 mm endmill at the spindle speed and feed rate of 14,000 rpm and 100 mm/min, respectively; (**d**) Photograph of bottom surface finished by 1 mm endmill after micromilling optimization; (**e**) Schematic illustration of Response Surface Methodology (RSM), based on face-centered, central composite design (CCD) with three levels per factor; (**f**) Contour plot showing the effect of feed rate and spindle speed on the response *Ra*. Black dots represent design points at final depth increment of 0.1 mm; (**g**) A schematic view of acetone vapor treatment setup; (**h**) A photograph of a micromilled channel after acetone vapor exposure (optimum) for 5 min at 25 °C; (**i**) A photograph of a micromilled channel after acetone vapor exposure (excessive) for 10 min at 25 °C.

Generally, to minimize the time and complexity of the micromachining optimization process, test studies should be performed on highly simplified models. In this study, as an example, a chromatographic column (Figure 3a) with microliter volume was fabricated using surface spotting to create a channel in a single toolpath.

For the micromilling process, a 3-axis CNC Mini-Mill/3 (Minitech machinery) was used. The uncoated carbide square-end endmill with a diameter of 1 mm and 2 flutes was used in the climb milling direction. Dry cooling and the chip removal process were accomplished using an air-blowing nozzle supplied with pressurized air (7 bar). The air nozzle tip, with an inner diameter of 8 mm, was positioned 5 cm away from the tip of the endmill and directed at a 45° angle to the tool axis.

The three controllable variables of feed rate, spindle speed, and final depth cutting increment were selected to minimize *Ra* as a response variable. The optimization process was performed by RSM based on face-centered, central composite design (CCD) with three levels per factor (Figure 3e). The total number of experiments in CCD design is calculated by Equation (4), where *i* is the number of variables. For the 3-factor system, *C_p_* equals 6. Thus, the total number of experiments equals 20.

(4) $$N=2^{i}\left(Factorial\;points\right)+2i\;\left(Axial\;points\right)+C_{p}(Central\;points)$$

The *Ra* was measured along the micromilled channel in three equally distanced regions (Figure 3a) using a surface profiler (Tencor Alphastep 200). The path length of each measurement in the chosen areas was 400 μm, one in the *x*-axis direction and one in the *y*-axis direction of the channel. The average of these two values was then calculated. In all 20 experiments, *Ra* values for the three sections were similar to each other, with percent deviations of less than 5% from the average value. The *Ra* of 20 experiments was then put into Design-Expert software version 11 (Stat-Ease, Minneapolis, MN, USA) as a response variable.

According to the in-house guiding protocol, the approximate range of spindle speed and feed rate for the endmill cutting tool with 0.5 mm ≤ *D_c_* ≤ 1.5 mm are about 8000–11,000 rpm and 300–750 mm/min, respectively. Figure 3b demonstrates an optical image of the milled surface with a 1 mm endmill at the spindle speed of 9000 rpm, feed rate of 300 mm/min and final cutting depth of 0.1 mm. The periodic features on the surface of the channel (Figure 3b) are formed by the cutting edges of the moving endmill in each cycle of cutting. In this study, considering the known approximate operability range for micromilling, a few initial experiments were performed for determining symmetrical parameter levels in RSM based on CCD. Considering the general process understanding (see Section 3.4.5), these experiments were implemented to find the upper range for spindle speed and the lower range for feed rate based on detecting tool breakage, gumming, or damage on the microfluidic structure. According to visual inspections, the spindle speed of 13,000 rpm (at feed rate of 100 mm/min) and feed rate of 50 mm/min (at spindle speed of 12,000 rpm) caused internal surface damage in the microfluidic structure (Figure 3c) and tool breakage, respectively. The minimum value for cutting depth was selected based on the in-house data regarding the maximum accuracy of the micromilling machine in the *z*-axis. The final range chosen for the operability window is presented in Table 1. The created design and the response data are presented in Table S1. If no previous information and experimental data were available, the steepest ascent method would be the best approach for defining the operating conditions in RSM optimization.

According to the statistical results, the first-order model with two-factor interaction (2FI) was shown to be the best fit. This model’s adjusted and predicted coefficients were 0.9418 and 0.8744, respectively, indicating a high correlation between the observed and predicted values. The 2FI was then modified to eliminate the parameters with a *p*-value above 0.1, including *C*, *AC*, and *BC* factors. The analysis of variance (ANOVA), shown in Table 2, suggests that the model, with a high *F*-value (*F* = 118.70) and a *p*-value below 0.0001, is significant and provides a good prediction for the experimental results. The high *p*-value for lack-of-fit also rejects the hypothesis of the significance of regression. The suggested 2FI model (Equation (5)) supports the general theoretical expectation that increasing spindle speed and decreasing feed rate reduce *Ra*. This outcome also supports the visual inspection results, which showed little or no presence of unfavorable effects of tool wearing and gumming during the whole optimization process. In the defined operating window, the feed rate was the dominant factor on *Ra*.

(5) Ra=1167.25+528.6A−290.60B−230.87AB

According to the results, the cutting depth did not have a notable effect on *Ra*. Nevertheless, this parameter’s value should not exceed a certain range, ~(0.5–1) ∗ *D_c_* for uncoated carbide, since increasing cutting depth increases the cutting force, a primary contributing factor to tool wearing and tool lifetime [39,40].

In Figure 3f, the contour plot shows the effect of feed rate and spindle speed on *Ra*. The curvature in the contour plot indicates the significance of the interactive responses of the feed rate and spindle speed factors. The best operation condition was predicted to be at a feed rate and spindle speed of 100 mm/min and 12,000 rpm, respectively, with the estimated *Ra* of about 0.578 μm. For confirmation, micromilling under these optimal operation conditions and cutting depth of 0.1 mm was performed. The measured value was about 0.534 μm (Figure 3d), which is significantly lower than those obtained using the recommended in-house protocol guideline (Figure 3b). Since the obtained *Ra*- before postprocessing- was acceptable for the microchromatographic column, no further RSM-based optimization, with adjusted parameter range for defining global optima, was performed.

The time factor was not considered during the optimization process since the fabricated PMMA structure was used as a master mold for the PDMS double-casting process. Besides, for the selected endmill tool, the total machining operation time under optimal conditions for creating a single cuboid channel with a length of 26 cm was only about 45 s.

#### 4.1.2. Postprocessing

The cleaning process of the microfabricated structure was done according to the protocol suggested by Matellan et al. [41]. First, the microstructure was washed with isopropanol (IPA) and then subjected to sonication in DI water for 2 min to remove any remaining chips or burrs [41]. Afterward, the microstructure was again rinsed with IPA and dried with pressurized air [41,42]. For polishing and reducing the *Ra* of the microstructure, acetone vapor treatment was carried out [41]. For this purpose, a Guyson Kerry ultrasonic bath (Skipton, North Yorkshire, UK) with a 300 W ultrasonic generator was employed. As depicted in Figure 3g, the PMMA microchannel was fixed with double-sided adhesive tape on the inner side of the plastic lid. Afterwards, at 25 °C and a frequency of 40 kHz, the ultrasonication time was optimized to polish the surface of the micromilled channel without causing excessive dissolution of the PMMA structure. Immediately after acetone vapor treatment, the microstructure was placed in an oven at a temperature of 70 °C to avoid creating cracks. Figure 3h,i demonstrate the milled channel under optimal (5 min) and excessive (10 min) acetone vapor treatment. The obtained result suggests that reducing the *Ra* up to 0.3 μm is possible with acetone vapor treatment without damaging the microstructure.

### 4.2. Strategies for Creating 3D Integrable Microsystems

**Figure 4:**
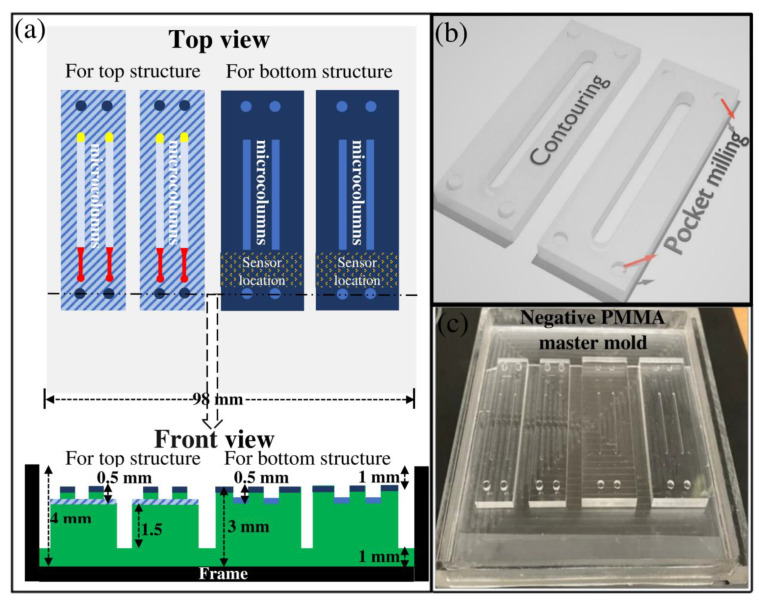
(**a**) Dimensional drawing of micromilled PMMA master mold for creating top structure and bottom structure of the final 3D PDMS microfluidic system; (**b**) Schematic outline of the experimental process to fabricate lego-like micromilled structure for easy alignment; (**c**) A photograph of micromilled PMMA master mold, with the negative features, inside the empty PMMA frame.

**Figure 5:**
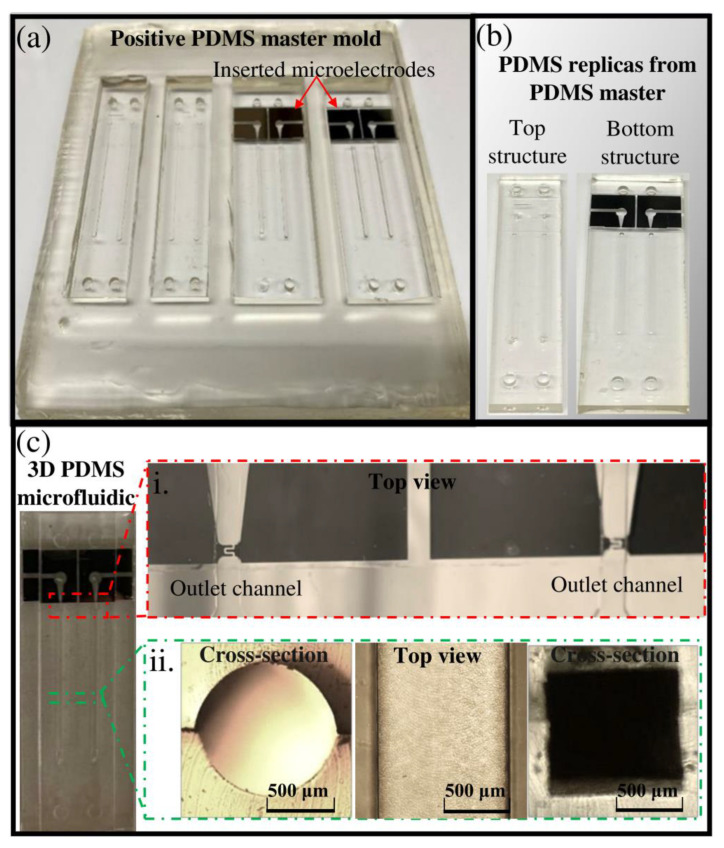
(**a**) A photo of positive PDMS master mold, made from negative PMMA master mold; (**b**) A photo of the top and bottom PDMS replica molds obtained from positive PDMS master mold; (**c**) Final PDMS microfluidic structure integrated with microelectrode after adherence of two PDMS replica molds (i) optical microscopy observations of the outlet channel, integrated with IDE (ii) cross-sectional and top view of microfluidic columns.

One of the advantages of the micromilling process is creating 3D microstructures with varying depths and heights. Although 3-axis micromilling has less design flexibility than 4-or 5-axis ones, complex features can still be made in this simple micromachining system. In this section, we defined a new strategy for creating 3D sensor-integratable microsystems (Figure 4 and Figure 5).

#### 4.2.1. Microfabrication Steps

In this study, a PMMA master mold with negative features (Figure 4) was used for the PDMS double-casting process. Implementing this method requires less surface area milling than the method in which PMMA serves as a positive master mold for PDMS casting. Besides, as mentioned in Section 3.3, various types of milling tools could be implemented to create different channel and chamber shapes. The details of double-casting techniques are described illustratively in a previous paper [43]. Briefly, we implemented a micromilling process for developing the negative PMMA master mold (Figure 4). Subsequently, this master mold was used to mold a positive PDMS master mold (1:5 ratio) at 70 °C for 3 h. The cured PDMS master mold was easily peeled off from the micromilled PMMA surface without any sticking problems (Figure 5a). Then, it was baked at 200 °C for 1 h to modify its surface chemistry. The positive PDMS master mold was then used for casting final PDMS replicas. The molded PDMS replicas were easily peeled off (Figure 5b) without leaving any residue on the top surface of the PDMS master mold.

#### 4.2.2. Negative PMMA Master Mold Micromilling Design

The detailed micromilling design of this case study is shown in Figure 4 and Table 3. In the development process of the micromilled PMMA master mold, we first made an empty frame, the black region in Figure 4a, from another thicker PMMA plate so that the micromilled PMMA master mold could be placed inside for PDMS molding (Figure 4c). The frame was created separately from the master mold to avoid milling the smooth surface of the PMMA original for making the walls for the frame. In this way, the general requirement for fine face milling to reduce surface roughness would be minimized, and operation time could be considerably shortened. Since the empty frame does not influence the surface quality of the final microstructure, a rapid rough pocket milling process with a 3-mm endmill was used to minimize the operation time. This fabricated frame can be used permanently for other PMMA master mold designs.

Figure 4a shows that two different micromilling surfaces were created to develop two separate top and bottom structures for the final 3D microfluidic system. During the negative PMMA master mold development by 3-axis micromilling, lego-like surfaces (Figure 4b) were created to significantly ease the alignment process. In the design process of the micromilled structure, a specific location in the bottom frame (Figure 4a) was assigned for sensor integration, which in this case were interdigitated planar microelectrodes (IDE). The design and fabrication of IDE, with a size of 174.2 × 174.2 μm, on a glass substrate, are described elsewhere [43]. As shown in Figure 4a, the width of the top structure is selected to be narrower than the bottom structure to create an accessible region for contact pads of IDE in the final PDMS microstructure (Figure 5c).

To develop all the mentioned features in the PMMA master mold, the micromilling operation required four steps with four different endmill sizes (Table 3). After each step, the milling process was stopped, surface and endmill quality were visually checked, and the next endmill was installed to the micromilling setup. Step 0 in Table 3, the dark blue color in Figure 4a, shows the region where milling was not required. Step 1 in Table 3, the grey color in Figure 4a, shows the bulk milling process of PMMA master mold with a 3 mm endmill. In step 2 (Table 3), for creating the primary microcolumns (Figure 4a), the feed rate and spindle speed values were selected based on the results obtained from Section 4.1.

The milling process condition of other regions was defined based on the decision-making toolbox described in Section 3.4. For instance, the size of the endmill and operation condition in the fine face milling process, step 3 in Table 3 (the hatched region in Figure 4a), were selected to create a balance between the minimization of surface roughness and operation time. Since this specific surface is the connecting and sealing region of the two final PDMS structures (Figure 5b), the surface roughness should be low enough to allow strong binding. Thus, endmills with smaller diameters, below 1 mm, with a slower feed rate and/or smaller stepover value, should be selected for fine milling. However, the endmill size and operation conditions should not dramatically increase the operation time and the possibility of tool breakage. In this study, an endmill with a diameter of 0.5 mm (step 3 in Table 3) demonstrated sufficient surface quality for surface binding when combined with acetone vapor postprocessing. To minimize the likelihood of tool wear and breakage of the endmill, a fast rough face milling process was performed in the previous step (step 2 in Table 3) with a bigger endmill size.

The total micromachining process time for creating the PMMA master mold was about 3 h 45 m. Considering that the micromilled PMMA is used for the purpose of molding, this is an acceptable length of time. Using the reported micromilled design (Figure 4), two 3D microfluidic structures can be created simultaneously. As shown in Figure S1, by using a bigger PMMA plate and increasing milling operation by a factor of about 2.5, the production of PDMS microfluidic structures could be increased to 5 in each PDMS double-casting process.

#### 4.2.3. Sensor Integration Strategy

In the case of sensor integration, before PDMS double-casting, the developed IDE was simply inserted and attached into the defined location in the positive PDMS master mold (Figure 5a). The presence of Van der Waals force between the microelectrode surface and PDMS master mold creates sufficient surface binding, preventing the diffusion of the PDMS liquid mixture between these two surfaces during the double-casting process. This binding is weak enough to easily peel off the final PDMS replicate containing the microelectrode inside it (Figure 5b). By creating an outlet channel in the opposite PDMS lego-like structure, the IDE with a direct electrical contact was easily integrated into the outlet channel of the 3D microfluidic system after binding the top and bottom PDMS structures (Figure 5c). The location of the IDE in the outlet channel (Figure 5c(i)), the top view and cross-sectional view of PDMS cuboid and circular columns (Figure 5c(ii)) indicate the accuracy of the alignment procedure.

## 5. Conclusions

In this study, first, a systematic methodology for developing high-quality thermoplastic-based molds for microfluidic structures is demonstrated. The impact of different milling parameters and postprocessing is presented for the case of a PMMA structure. A case study for optimizing *Ra* for fabricating a cuboid microcolumn is presented using RSM. The results show the degree of impact of feed rate, spindle speed, and postprocessing on the finished *Ra* with a final value of 0.223 μm. In the second part of the study, a specific micromilling design was developed for creating 3D microfluidic structures, which offers the possibility of easy alignment and integration of microstructures and microsensors.

## Figures and Tables

**Table 1:** Values of independent variables at high and low levels.

Process Parameters	Coded Symbol	Levels	
		Coded Low (−1)	Coded High (+1)
Feed rate (mm/min)	A	100	300
Spindle speed (rpm)	B	6000	12,000
Final cutting depth (mm)	C	0.05	0.15

**Table 2:** ANOVA table for reduced 2FI model.

Source	Sum of Squares [10^6^]	DF	Mean Square [10^6^]	*F*-Value	*p*-Value
Model	4.065	3	1.355	118.70	<0.0001
*A*-Feed rate	2.794	1	2.794	244.76	<0.0001
*B*-Spindle speed	0.8445	1	0.8445	73.97	<0.0001
*AB*	0.4264	1	0.4264	37.35	<0.0001
Residual	0.1827	16	0.01141603		
Lack of Fit	0.1271	11	0.01155905	1.04	0.5183
Pure Error	0.05550683	5	0.01110137		
Cor Total	4.248	19			

**Table 3:** Implemented micromachining process for developing PMMA master mold with negative features (Figure 4a).

Step	Endmill (2-Flute)	Milling Type	Cutting Depth (mm)	Spindle Speed (rpm)	Feed Rate (cm/s)	Step Over (mm)	Approximate Operation Time (min)
0	Nonmilled	N/A 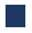	0	N/A	N/A	0	0
1	3 mm	Contouring 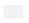	2	10,000	600	0.3	44
2	1 mm	Face milling (rough) 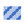	0.5	12,000	300	0.3	35
		Pocket milling 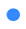	0.5	12,000	100	0.3	3
		Face spotting-Microcolumn 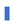	0.5	12,000	100	-	2
		Face spotting-Microcolumn 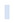	0.5 *	12,000	100	-	2
3	0.5 mm	Face milling (fine) 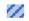	0 *	16,000	75	0.3	132
		Pocket milling (rough) 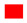	0.3 *	16,000	75	0.2	2
4	0.1 mm	Pocket milling (fine) 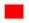	0.3 *	16,000	50	0.3	5
		Pocket milling (fine) 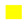	0.1 *	16,000	50	0.2	2

* The cutting depth values are based on the new surface area created by the face milling (rough), with 1 mm Endmill, in step 2.

## Data Availability

Data is contained within the article.

## Supplementary Materials

The following are available online at https://www.mdpi.com/article/10.3390/mi13010006/s1, Figure S1: A photo of the micromilled PMMA master mold, with the negative features, with the possibility of parallel fabrication of five 3D PDMS microfluidic structures. Table S1: The created design and response data, surface roughness, in micromilling optimization.
