# Supplementary material for: A Systematic Approach for Developing 3D High-Quality PDMS Microfluidic Chips Based on Micromilling Technology
Source: Micromachines (Basel). 2021 Dec 22;13(1):6. doi: 10.3390/mi13010006 (PMC8779272; doi:10.3390/mi13010006)

Supplementary Material

A systematic approach for developing 3D high-quality PDMS microfluidic chips based on micromilling technology

Amin Javidanbardan[1,2], Ana M. Azevedo[1,2],Virginia Chu[3], João P. Conde[2,3*]*

[1]IBB – Institute for Bioengineering and Biosciences, Instituto Superior Técnico, Universidade de Lisboa, Lisbon, Portugal

[2]Department of Bioengineering, Instituto Superior Técnico, Universidade de Lisboa, Lisbon, Portugal

[3]Instituto de Engenharia de Sistemas e Computadores – Microsistemas e Nanotecnologias (INESC MN) and IN – Institute of Nanoscience and Nanotechnology

**Corresponding authors.*

Figure S1. A photo of the micromilled PMMA master mold, with the negative features, with the possibility of parallel fabrication of five 3D PDMS microfluidic structures.

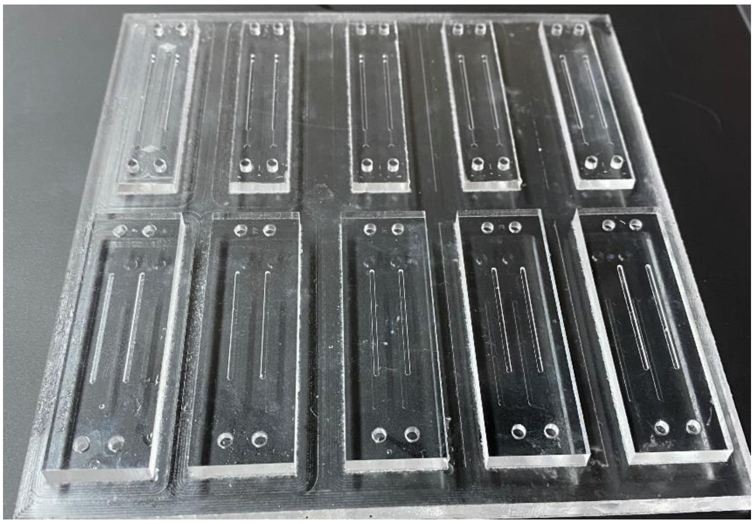

Supplement: Supplementary file 1 [file micromachines-13-00006-s001.zip › Supplementary Material Figure S1.pdf]
