# Supplementary material for: A Systematic Approach for Developing 3D High-Quality PDMS Microfluidic Chips Based on Micromilling Technology
Source: Micromachines (Basel). 2021 Dec 22;13(1):6. doi: 10.3390/mi13010006 (PMC8779272; doi:10.3390/mi13010006)
Supplement: Supplementary file 1 [file micromachines-13-00006-s001.zip › Supplementary Material TableS1.pdf]

Supplementary Material

A systematic approach for developing 3D high-quality PDMS microfluidic chips based on micromilling technology

Amin Javidanbardan[1,2], Ana M. Azevedo[1,2], Virginia Chu[3], João P. Conde[2,3*]*

[1]*IBB – Institute for Bioengineering and Biosciences, Instituto Superior Técnico, Universidade de Lisboa, Lisbon, Portugal*

[2]*Department of Bioengineering, Instituto Superior Técnico, Universidade de Lisboa, Lisbon, Portugal*

[3]*Instituto de Engenharia de Sistemas e Computadores – Microsistemas e Nanotecnologias (INESC MN) and IN – Institute of Nanoscience and Nanotechnology*

**Corresponding authors.*

Table S1. The created design and response data, surface roughness, in micromilling optimization

Std	Run	A-Feed rate (mm/min)	B-Spindle speed (rpm)	C-Final depth increment (mm)	Surface roughness (nm)
7	1	100	12000	0.15	507
5	2	100	6000	0.15	605
1	3	100	6000	0.05	622
8	4	300	12000	0.15	1150
10	5	300	9000	0.1	1620
16	6	200	9000	0.1	1085
20	7	200	9000	0.1	1073
6	8	300	6000	0.15	2126
17	9	200	9000	0.1	1203
9	10	100	9000	0.1	793
4	11	300	12000	0.05	1180
18	12	200	9000	0.1	1150
13	13	200	9000	0.05	1173
2	14	300	6000	0.05	2254
19	15	200	9000	0.1	1328
14	16	200	9000	0.15	1042
12	17	200	12000	0.1	987
15	18	200	9000	0.1	1290
11	19	200	6000	0.1	1640
3	20	100	12000	0.05	517
